# Structural comparison of tRNA m^1^A58 methyltransferases revealed different molecular strategies to maintain their oligomeric architecture under extreme conditions

**DOI:** 10.1186/1472-6807-11-48

**Published:** 2011-12-14

**Authors:** Amandine Guelorget, Pierre Barraud, Carine Tisné, Béatrice Golinelli-Pimpaneau

**Affiliations:** 1 Laboratoire d'Enzymologie et Biochimie Structurales, Centre de Recherche de Gif, CNRS, 1 avenue de la Terrasse, 91190 Gif-sur-Yvette, France; 2 CNRS, UMR 8015, Laboratoire de Cristallographie et RMN biologiques, 4 avenue de l'Observatoire 75006 Paris, France; 3 Université Paris Descartes, Sorbonne Paris Cité, UMR 8015, Laboratoire de Cristallographie et RMN biologiques, 4 avenue de l'Observatoire 75006 Paris, France; 4 Institute of Molecular Biology and Biophysics, ETH Zurich, 8093 Zürich, Switzerland

## Abstract

**Background:**

tRNA m^1^A58 methyltransferases (TrmI) catalyze the transfer of a methyl group from S-adenosyl-L-methionine to nitrogen 1 of adenine 58 in the T-loop of tRNAs from all three domains of life. The m^1^A58 modification has been shown to be essential for cell growth in yeast and for adaptation to high temperatures in thermophilic organisms. These enzymes were shown to be active as tetramers. The crystal structures of five TrmIs from hyperthermophilic archaea and thermophilic or mesophilic bacteria have previously been determined, the optimal growth temperature of these organisms ranging from 37°C to 100°C. All TrmIs are assembled as tetramers formed by dimers of tightly assembled dimers.

**Results:**

In this study, we present a comparative structural analysis of these TrmIs, which highlights factors that allow them to function over a large range of temperature. The monomers of the five enzymes are structurally highly similar, but the inter-monomer contacts differ strongly. Our analysis shows that bacterial enzymes from thermophilic organisms display additional intermolecular ionic interactions across the dimer interfaces, whereas hyperthermophilic enzymes present additional hydrophobic contacts. Moreover, as an alternative to two bidentate ionic interactions that stabilize the tetrameric interface in all other TrmI proteins, the tetramer of the archaeal *P. abyssi *enzyme is strengthened by four intersubunit disulfide bridges.

**Conclusions:**

The availability of crystal structures of TrmIs from mesophilic, thermophilic or hyperthermophilic organisms allows a detailed analysis of the architecture of this protein family. Our structural comparisons provide insight into the different molecular strategies used to achieve the tetrameric organization in order to maintain the enzyme activity under extreme conditions.

## Background

Extremophiles are microorganisms that are found in environments of extreme temperature (-2°C to 15°C, 60-110°C), ionic strength (2-5 M NaCl) or pH (< 4, > 9). They are source of enzymes with extreme stability (extremozymes). Understanding the origin of this stability at a molecular level is very attractive as extremozymes are stable and active under conditions previously thought to be incompatible with biological materials. Only represented by bacterial and archaeal species, hyperthermophiles grow optimally at temperatures above 80°C [1]. Some enzymes from hyperthermophiles are active at temperatures as high as 110°C and even above [2]. To clarify, the term thermostability refers to the preservation of the unique chemical and three-dimensional structure of a polypeptide chain under extreme temperature conditions.

The comparison of mesophilic and thermostable homologous proteins has revealed some important factors that contribute to the remarkable stability of thermoenzymes. Previously reported studies aiming at establishing the origin of thermostability have compared the sequence and/or the structure of homologous proteins from thermophiles and mesophiles. Concerning the primary sequence, different characteristics have been identified as contributors to stability. First, significant changes in the amino-acid composition between mesophilic and thermophilic proteins have been described. Charged and hydrophobic residues are often over-represented in thermophilic proteins [3-5]. A higher Proline content, related with higher rigidity of the backbone has also been reported [6,7]. Long and flexible loops tend to be absent in thermostable proteins and are often replaced by short and rigid ones [8-10]. Different structural features have also been shown to contribute to protein thermostability, such as an increased number of hydrogen bonds, more ionic interactions, greater hydrophobic interactions, a more compact and rigid packing, and the presence of disulfide bridges [11-14]. Importantly, these studies revealed that there is no single universal mechanism that promotes stability, and the molecular mechanisms behind thermostability can vary from one protein to the other [1,11,12].

Numerous chemical modifications occur after transcription during the tRNA maturation process [15]. tRNA modification enzymes from extremophiles have not been so far the subjects of detailed structural analysis aiming at understanding the molecular basis of their stability. Actually, only thirteen post-transcriptional tRNA base modifications are conserved among the three domains of life, and twenty of them are common to bacteria and archaea [16]. Here, we compare the available crystal structures of TrmI methyltransferases (MTases) that methylate the N1 atom of adenine at position 58 in the T-loop of tRNA. m^1^A58 is one of the modifications present in the three domains of life although it is not frequently found in bacteria. It has been proposed that the presence of this positively charged modified nucleotide, which is located on the outer edge of the molecular tRNA structure, is important for the tRNA tertiary structure and/or for recognition by its partner proteins. In the yeast *Saccharomyces cerevisiae*, m^1^A58 is essential for cell growth under normal conditions, as shown by the non-viability of mutants defective in N1-methylation of A58 in initiator tRNA [17,18], whereas in the bacterium *Thermus thermophilus*, the TrmI enzyme is required for cell growth at high temperatures [19].

Although S-Adenosyl-L-Methionine (SAM) MTases displaying a Rossmann-like fold are mostly monomeric [20], the TrmI proteins share a conserved tetrameric quaternary structure both in solution [19,21-24] and in the crystals [25-27]. This architecture is unique among the tRNA modification enzymes characterized up to now. In bacteria and archaea, the enzyme consists of a tetramer formed by identical subunits of about 30 kDa. In contrast, the yeast [24] and human tRNA m^1^A58 MTases [23] are hetero-tetrameric enzymes composed of two different subunits encoded by the *TRM6 *and *TRM61 *genes. It has been proposed that both subunits of eukaryotic tRNA m^1^A58 MTases evolved from a common ancestor through gene duplication and divergent evolution [28]. Amino acid substitutions in either subunit prevent the yeast enzyme from binding to tRNA^Met^_i_, indicating that each subunit contributes to tRNA recognition [24]. In the case of the homo-tetrameric *T. thermophilus *TrmI, noncovalent mass spectrometry analysis showed that the enzyme binds to its tRNA substrate as a tetramer and is able to bind up to two tRNAs per tetramer [26]. This suggests that the structurally identical subunits have non-equivalent roles within the tetrameric structure, which is reminiscent of the case of homo-tetrameric archaeal tRNA splicing enzymes [29] and O-phosphoseryl-tRNA:selenocysteinyl-tRNA synthase [30]. This would provide an explanation for the existence of both homo- and heterotetramers of TrmI proteins depending on the organism.

In the present report, we have performed comparative studies of the available crystal structures of TrmI proteins to highlight their common properties and shed light on the different structural factors that might explain the stability of TrmI enzymes from extremophiles. We have first compared the TrmI monomers and examined the different mechanisms that can contribute to the thermal stability of the subunit structure. Secondly, since the subunits of thermostable oligomeric enzymes are generally more tightly assembled than in less stable homologous species [31,32], we have analyzed and compared the inter-subunit contacts in the various crystal structures. Interestingly, our study revealed that different strategies at the level of the inter-subunit contacts have been developed to stabilize the TrmI proteins from thermophilic and hyperthermophilic organisms. The key to achieve TrmI activity under extreme conditions of life appears to lie in the preservation of the tetrameric organization.

## Results and discussion

### Structural comparison of TrmI proteins

#### The archaeal and bacterial m^1^A58 MTases have similar size and architecture

The crystal structures of three bacterial and one archaeal TrmIs have previously been reported (Table 1): from *Mycobacterium tuberculosis*, a mesophilic bacterium (_Mt_TrmI, initially called Rv2118c) [22,25], *Thermus thermophilus*, a thermophilic bacterium (_Tt_TrmI)[26], *Aquifex aeolicus *(PDB code 2YVL, _Aa_TrmI), a hyperthermophilic bacterium and *Pyroccocus abyssi*, a hyperthermophilic archaeon (_Pa_TrmI) that lives in an environment of extreme pressure [27]. Moreover, a search in the Dali database reveals that the PDB code 1O54, a putative SAM-dependent O-MTase from the thermophilic bacterium *Thermotoga maritima, *is inaccurately annotated and corresponds to the m^1^A58 MTase (_Tm_TrmI). Finally, the PDB code 2B25, annotated as a human putative 1-methyladenosine MTase, corresponds to the product of the TRM61 gene (_Hs_TrmI-61), the SAM-binding subunit that composes the hetero-tetramer of m^1^A58 MTase in eukaryotes and shows extensive sequence similarity to the bacterial and archaeal enzymes [23]. These organisms, with structurally characterized TrmI proteins, display very different optimal growth temperatures (Table 1). These TrmI proteins show sequence identity ranging from 24.8% to 40.8%, the bacterial proteins from *M. tuberculosis *and *T. thermophilus *being the most similar and the two hyperthermophilic proteins (*P. abyssi *and *A. aeolicus*) the most dissimilar (Additional File 1, Table S1). Except otherwise stated, the residue numbering for *T. maritima *is used throughout the text.

**Table 1:** Crystal structures of TrmI proteins available in the PDB.

Name	Species (domain of life)	Optimal growth temperature ^a ^(°C)	PDB code	Resolution (Å)	Space group	ligand	**Number of monomers in the asymmetric unit**^**b**^
_Hs_TrmI-61	*H. sapiens *(**E**)	37 (**M**)	2B25	2.5	C222_1_	SAM	2

_Mt_TrmI	*M. tuberculosis *(**B**)	37 (**M**)	1I9G	1.98	I222	SAM	1

_Tt_TrmI	*T. thermophilus *(**B**)	70 (**T**)	2PWY	1.7	C222_1_	SAH	2

_Tm_TrmI	*T. maritima *(**B**)	77-80 (**T/HT**)	1O54	1.65	I222	-	1

_Aa_TrmI	*A. aeolicus *(**B**)	85-96 (**HT**)	2YVL	2.2	P2_1_2_1_2_1_	SAM	4

_Pa_TrmI	*P. abyssi *(**A**)	100-103 (**HT, Ba**)	3LHD 3LGA 3MB5	2.6 2.05 1.6	P2_1_2_1_2_1_ P3_1_ I222	SAH SAH SAM	4 4 1

^a ^When different optimal growth temperatures have been reported, a range of temperature is given.

^b ^The biological assembly predicted by PISA is a tetramer in all cases.

A: Archaea, B: Bacteria, E: Eukarya

Ba: Barophile, M: Mesophile, T: Thermophile, HT: Hyperthermophile

SAM: S-adenosyl-L-methionine

SAH: S-adenosyl-L-homocysteine

**Figure 1:**
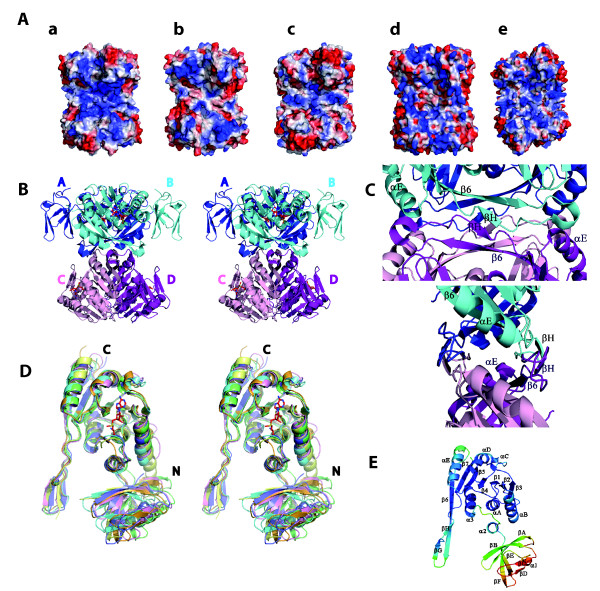
**Structural comparison of TrmIs**. **A **The electrostatic surfaces of each TrmI protein were determined using PYMOL/APBS and are colored by the electrostatic potential. The values of surface potential range from -72 kT/e (red) to +72 kT/e (blue). **a **_Mt_TrmI **b **_Tt_TrmI **c **_Tm_TrmI **d **_Aa_TrmI **e **_Pa_TrmI. **B **Stereoview of the tetrameric architecture of _Aa_TrmI with the SAM ligand in red sticks. The structure is shown with a 45 degree rotation around the vertical symmetry axis compared to Figure 1A. **C **Closer view showing the tetrameric interface in two orthogonal orientations. The main secondary structure elements are labeled. **D **Stereo view of the superimposition of the monomer from the six TrmI structures. The Cαs of residues 85 to 263 corresponding to the C-terminal domain (_Tm_TrmI numbering) are superposed. _Pa_TrmI (PDB code 3MB5), _Aa_TrmI, _Hs_TrmI, _Mt_TrmI, _Tm_TrmI and _Tt_TrmI are drawn in green, cyan, yellow, pink, orange and purple, respectively, and SAM in red sticks. The N-and C-termini are indicated by the letters N and C, respectively. **E **View of one monomer of _Pa_TrmI (PDB code 3MB5) labeled with the secondary structure elements and with the polypeptide chain colored according to the B-factors (low B-factor in blue, high B-factor in red).

The TrmI proteins show a tetrameric organization in the crystals as it was shown in solution [19,21-24]. The crystallographic asymmetric unit consists of one, two or four subunits of the protein (Figure 1, Table 1) and the tetramer is assembled from the monomers using either the crystallographic or non-crystallographic symmetry. The four subunits of the homo-tetrameric TrmI proteins are related by a four-fold symmetry. Two opposite sides of the tetramer show positively charged grooves, wide enough to accommodate an A-form RNA helix [26], that likely bind the tRNA substrate (Figure 1A). Interestingly, the electrostatic surfaces are not similar in all TrmI proteins, _Tm_TrmI showing a less positive surface than the four other proteins and _Mt_TrmI and _Pa_TrmI having the most positive surface (Figure 1A). Based on the characteristics of the different monomer-monomer interfaces, the tetramer can be described as a dimer of two tightly assembled dimers (A/B and C/D), which interact back to back as shown in Figure 1B. The two protruding antiparallel β-strands at the C-terminus of each monomer contribute significantly to the tetrameric assembly (Figure 1C).

_Mt_TrmI, _Tm_TrmI and _Pa_TrmI (space group I222) contain one TrmI monomer in the asymmetric unit and the tetramer is generated using the crystallographic symmetry. In _Aa_TrmI and in the two crystal forms of _Pa_TrmI in complex with SAH, the crystallographic asymmetric unit contains a full tetramer and there are similar relationships between the monomers except that all axes are non-crystallographic. The four monomers display an rmsd between equivalent Cα atoms of less than 0.27 Å, 0.66 Å and 0.05 Å for _Aa_TrmI, _Pa_TrmI (space group P2_1_2_1_2_1_) and _Pa_TrmI (space group P3_1_), respectively. In _Tt_TrmI and _Hs_TrmI, the asymmetric unit contains the tight A/B dimer and the A/D dimer, respectively, in which the two monomers are related by the non-crystallographic 2-fold axis. The two monomers are almost identical with an rmsd between equivalent Cα atoms of 1.04 Å and 0.36 Å for _Tt_TrmI and _Hs_TrmI, respectively. The full tetramer of _Tt_TrmI is generated by proper crystallographic 2-fold symmetry. Although a homo-tetramer of _Hs_TrmI-61 can also be formed using the 2-fold crystallographic symmetry, it is not biologically relevant because _Hs_TrmI, in contrast to bacterial and archeal TrmIs, is a hetero-tetramer. The fact that the asymmetric unit of the _Hs_TrmI-61 crystal consists of the A/D dimer is consistent with the modeling of the full yeast TrmI structure [24], and strongly suggests that in eukaryotic TrmIs, the A/B and A/C dimers are formed by two different subunits (TrmI-6 and TrmI-61). Therefore, in eukaryotes, the dimeric and tetrameric contacts are formed only between different subunits. The dimers and tetramers of all TrmIs are very similar, and the tetramers of all structures can be superimposed with rmsd of 1.65-3.2 Å (Additional File 1, Table S2A). Therefore, there is no rigid body rearrangement of the monomers between the TrmI proteins, which, in some cases [33], was shown to be a factor contributing to thermal stability.

#### Structural comparison of the TrmI monomers shows mobility of the N-terminal domain relative to the C-terminal catalytic domain

**Figure 2:**
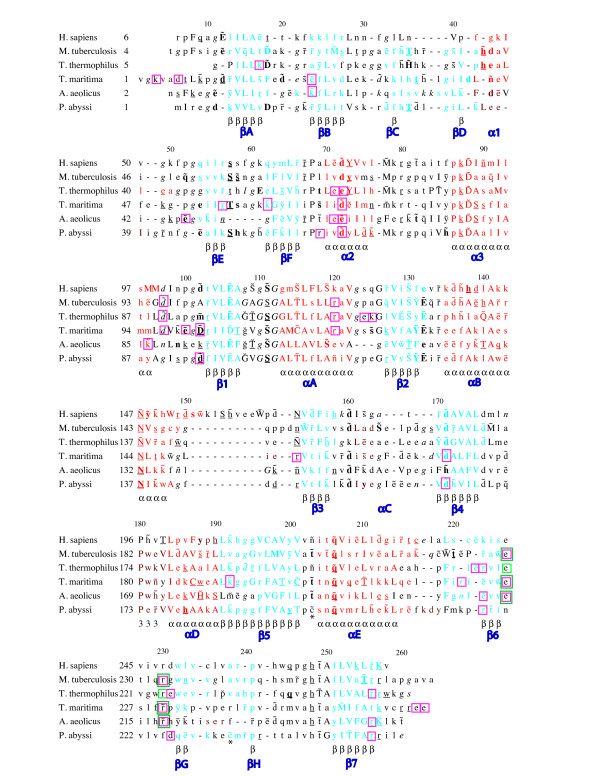
**Structural alignment of the amino acid sequences of TrmI proteins with known three-dimensional structures using *JOY ***[52]. Solvent inaccessible residues in the monomer are shown in upper-case letters. Residues belonging to α-helices, β-strands and 3-10 helices are shown in red, blue and maroon, respectively. The consensus secondary structure and its numbering in _Tm_TrmI (PDB sequence, residue 79 is missing) is shown underneath and above the sequences, respectively. In addition, numbering for each protein (PDB sequence) is indicated on the left of the sequence. Residues with positive phi torsion angle are shown in italic and cis peptide by a breve over the amino acid concerned. Residues that hydrogen bond to main-chain amide and to main-chain carbonyl are indicated in bold and underlined characters, respectively. Hydrogen bond to other side chain is indicated by a tilde (~) over the amino acid concerned. Residues involved in salt bridges at the dimeric and tetrameric interfaces are enclosed in pink and green squares, respectively. The cysteines of _Pa_TrmI, which are involved in inter-monomer disulfide bridges, are indicated by a star. The secondary structure elements are labeled according to the nomenclature defined by Schluckebier et al. [54]. For the C-terminal catalytic domain, β-strands and α-helices are associated with numbers and letters, respectively, whereas the reverse is used for the N-terminal auxiliary domain.

**Figure 3:**
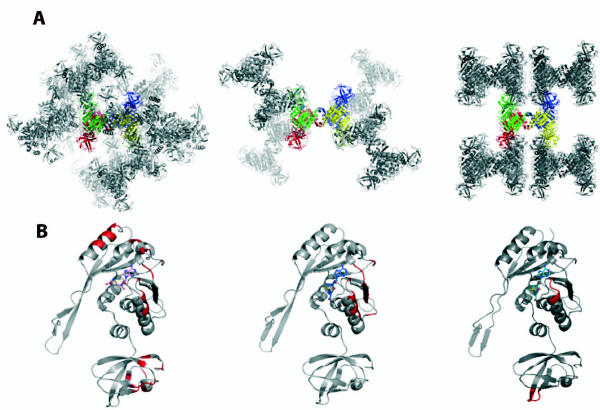
**Crystallographic contacts in the various _Pa_TrmI structures**. **A **Different crystal packing of _Pa_TrmI in the different crystal forms (left P2_1_2_1_2_1_, middle P3_1_, right I222). **B **The regions involved in the crystallographic contacts are shown in red, SAH/SAM in sticks.

The monomer structures of TrmI proteins with known three-dimensional structure are closely similar, with rmsd between 1.34 and 2.70 Å and Q-scores between 0.41 and 0.59 (Additional File 1, Table S2B; Figure 1(D)). One monomer is formed by two domains: a catalytic C-terminal domain that binds the SAM/SAH cofactor with a Rossmann-like fold characteristic of SAM-dependent MTases, and a smaller N-terminal domain with a β-structure. A structure-based multiple sequence alignment and secondary structure assignment for the TrmI monomers are shown in Figure 2. The N-terminal domain (residues 1-67) contains one helix (α1) and six β-strands (βA to βF). The C-terminal domain (residues 68-263) contains seven α-helices and eight β-strands, of which strands β6, βG and βH form an antiparallel β-sheet involved in the tetramer formation. Compared to the other enzymes, _Hs_TrmI-61 has a 10-residue insertion in the turn between αB and β3. The similarity of the structures extends even higher when only the catalytic domains are superposed (Figure 1(D)), revealing a slightly different relative orientation of the N-terminal and catalytic domains in the TrmIs studied. Moreover, comparison of the four monomers in the structure of _Pa_TrmI in the P2_1_2_1_2_1 _space group, in which SAH adopts two different conformations [27], reveals also mobility of the N-terminal domains relative to the tetramer core formed by four catalytic domains. Indeed, the four monomers in the asymmetric unit display a pair-wise rmsd of less than 0.66 Å comparing 253 pairs of Cα atoms, whereas the superposition of the catalytic domains alone gives an rmsd of less than 0.36 Å. The higher B-factors of the N-terminal domain also indicate its mobility relative to the tetramer core (Figure 1 (E)). It is possible that the mobility of the N-terminal domain may be critical to the activity of the enzyme and that a hinged movement may occur upon tRNA binding, as observed for other RNA modifying enzymes [34]. However, although the crystallographic contacts of _Pa_TrmI in the three different space groups are different (Figure 3), the relative orientation of the catalytic and N-terminal domains remains unchanged, which suggests the existence of a preferred conformation of the protein in the absence of tRNA.

### Factors responsible for the stability of TrmI proteins under extreme conditions

Structural comparison of homologous enzymes from thermophiles and mesophiles suggests that thermostability originates from several factors [11]. More ion pairs and hydrogen-bonding interactions, reduced exposure of hydrophobic surface, tighter hydrophobic packing of the protein core, reduction in the number and volume of cavities, as well as improved inter-subunit contacts within oligomeric proteins contribute to increasing thermostability [8,31,32,35-39]. These various factors were examined in the case of the TrmI proteins.

#### Sequence comparison

Usually, proteins from thermophiles contain more charged and hydrophobic amino acids residues at the expense of polar ones [1,40]. Additionally, analysis of mesophilic and thermophilic proteins has previously pointed out the tendency towards shorter (even absent) loop regions in thermophilic organisms, which correlates with their compactness [8,41]. The number of alanine residues and aromatic amino acids is not higher in thermostable TrmIs compared to the less-stable ones (Additional File 1, Table S3). A higher number of prolines increases the backbone rigidity and has been shown to contribute in some cases to protein thermostability [42]. Here, the content of proline residues (between 3.6 and 6.3%; Additional File 1, Table S3) did not reveal outstanding differences between the various TrmI proteins, indicating that reduction of the backbone flexibility is not a factor contributing importantly to the thermostability of TrmI proteins. Noticeably, _Aa_TrmI displays some loop shortenings (Figure 2) and the smallest surface area among the structurally characterized TrmIs (Table 2), two features that could contribute to the thermal stability of _Aa_TrmI.

**Table 2:** TrmI molecular volumes and cavity volumes.

Organism	Cavity volumes^a^		Compactness		Protein Volume (A^3^)				
	**Total cavity volumes (A**^**3**^**)**	**number of cavities**	**Surface area (Å**^**2**^**)**	**Surface/Volume ratio (Å**^**-1**^**)**	**Monomer *a***	**Dimer *b *A/B**	**Tetramer *c***	**Δ 2 = *b - 2a***	**Δ 4 = *c - 4a***

*M. tuberculosis*	1 501	2	41130	0.39	26 460	52 970	105 900	+ 50	+ 60

*T. thermophilus*	1 182	2	39120	0.38	25 870	51 700	103 100	-40	- 380

*T. maritima*	2 154	3	41210	0.37	27 930	55 860	111 700	0	- 20

*A. aeolicus*	1 697	3	38680	0.36	27 000	53 955	107 900	- 45	- 100

*P. abyssi*									
3MB5	2 036	3	41880	0.38	27 420	54 820	109 600	-20	- 80
3LGA					27 330	54 635	109 200	-30	- 130
3LHD					26 440	52 840	105 700	-45	- 70

^a ^calculated for TrmI monomers without ligands

#### H-bonds and salt bridges within one TrmI monomer

Increased ion-pairing and hydrogen bonding interactions are factors employed by thermophiles to stabilize their proteins at extreme temperatures. The participation of these interactions in the stability of the monomer was analyzed (Additional File 1, Table S4). The two hyperthermophilic TrmI proteins have the highest number of intra-monomer salt bridges (18 and 20). However, these interactions are not more numerous in the case of the thermophilic TrmI proteins compared to the mesophilic ones. Surprisingly, there are only 6 intra-monomer salt bridges in _Tt_TrmI compared to 11 to 20 in the other TrmI proteins. The number of H-bonds per monomer (203 to 231) is similar in all TrmI proteins.

#### Compactness

Although oligomerization has been identified as one of the ways to achieve thermostability of proteins in thermophilic organisms [43], both mesophilic and thermophilic TrmI proteins are organized as tetramers. This quaternary structure therefore does not result from adaptation to high temperatures but is important for binding the tRNA substrates and for catalytic activity [19,21-24,26].

Although the fine interactions between monomers can obviously be modulated by the flexibility of residues located at the structures interfaces, the analysis of the crystallographic coordinates is likely to unveil several features of the quaternary structure characteristic of the thermozymes. To investigate whether TrmI extremozymes are more tightly packed than their mesophilic homologs, the surface areas and the molecular protein volumes of the monomer, A/B dimer and tetramer for each homo-tetrameric protein were calculated (Table 2). All five enzymes have similar surface areas. The monomer protein volume and the number and volume of cavities do not decrease with higher stability of the protein. However, the difference between the volume of the tetramer and the volume of four monomeric units (Δ4 = c-4a, Table 2) is negative for all thermophilic and hyperthermohilic TrmIs, indicating that the protein density increases upon tetramerization. In contrast, this number is positive for the mesophilic _Mt_TrmI protein. Yet, the amount of volume contraction is not directly dependent of the optimal growth temperature of the thermophilic protein. Similarly, the contraction upon dimer formation (Δ2 = b-2a, Table 2) is negative for both thermophilic and hyperthermophilic proteins. This reflects a tight packing of the hyperthermophilic A/B dimers. The surface to volume ratio is another way to measure the compactness (Table 2). Interestingly, this factor decreases as the thermostability of the TrmI protein increases, except for _Pa_TrmI. This might reflect the particularity of this enzyme to possess intermolecular disulfide bridges (see below). Again with the exception of the *P. abyssi *enzyme, increased buried surface areas within the A/B dimer and within the tetramer (Table 3) correlate with increased thermostability of the TrmI proteins. Indeed, the mesophilic protein has a smaller buried surface area compared to the thermophilic and hyperthermophilic ones. These features related to the oligomerization of the proteins suggested some critical differences at the interfaces of the TrmI monomers and led us to analyze in detail the network of interactions at these interfaces.

**Table 3:** Dimeric and tetrameric contacts in TrmI proteins.

	A/B dimer							A/C dimer							Tetrameric assembly		
**structures**	**N° of interfacing residues in both partners**	**Interface area (Å**^**2**^**)**	**Buried surface area (Å**^**2**^**)**	**N**_**HB**_^**1**^	**N**_**SB**_^**2**^	**N**_**vdW**_^**3**^	**hydrophobic *P*-value**^**4**^	**N° of interfacing residues in both partners**	**Interface area (Å**^**2**^**)**	**Buried surface area (Å**^**2**^**)**	**N**_**HB**_^**1**^	**N**_**SB**_^**2**^	**N**_**vdW**_^**3**^	**hydrophobic *P*-value**^**4**^	**Buried surface area (Å**^**2)**^	**ΔGint**^**5 **^**kcal/mol**	**ΔGdiss**^**6 **^**kcal/mol**

*M. tuberculosis*	76+75	2378.4	4760	28	6 (0)	30	0.299	23+23	788.7	1580	10	4 (2)	10	0.22	13160	-69.5	24.7

*T. thermophilus*	95+62	2478.1	4960	21	18 (5)	30	0.776	22+22	731.3	1460	12	4 (2)	13	0.423	13390	-25.9	20.8

*T. maritima*	87+87	3150.2	6160	24	36 (4)	103	0.356	24+24	879.5	1810	10	4 (2)	22	0.668	16750	-47.9	3.0

*A. aeolicus*	65+65	2596.3	5200	34	14 (0)	44	0.059	22+24	856.5	1710	12	4 (2)	4	0.416	14410	-54.7	15.1

*P. abyssi*	65+65	2311.5	4620	17	14 (6)	30	0.006	24+24	775.6	1550	12	0	10	0.069	12840	-86.6	41.0

^1^Number of H-bonds less than 3.2 Å across the interface (per monomer).

^2^Number of salt bridges less than 3.6 Å across the interface (per monomer). The number of bidentate ionic interactions is indicated in parentheses.

^3^Number of van der Waals interactions less than 4.2 Å (per monomer)

^4^defined as the probability to find a same-area patch on protein surface that would be more hydrophobic than the interface

^5^solvation free energy gain upon formation of the assembly

^6^free energy difference between dissociated and associated states

#### Inter-monomer H-bonds, salt bridges and hydrophobic contacts contributing to the TrmI tetramer architecture

**Figure 4:**
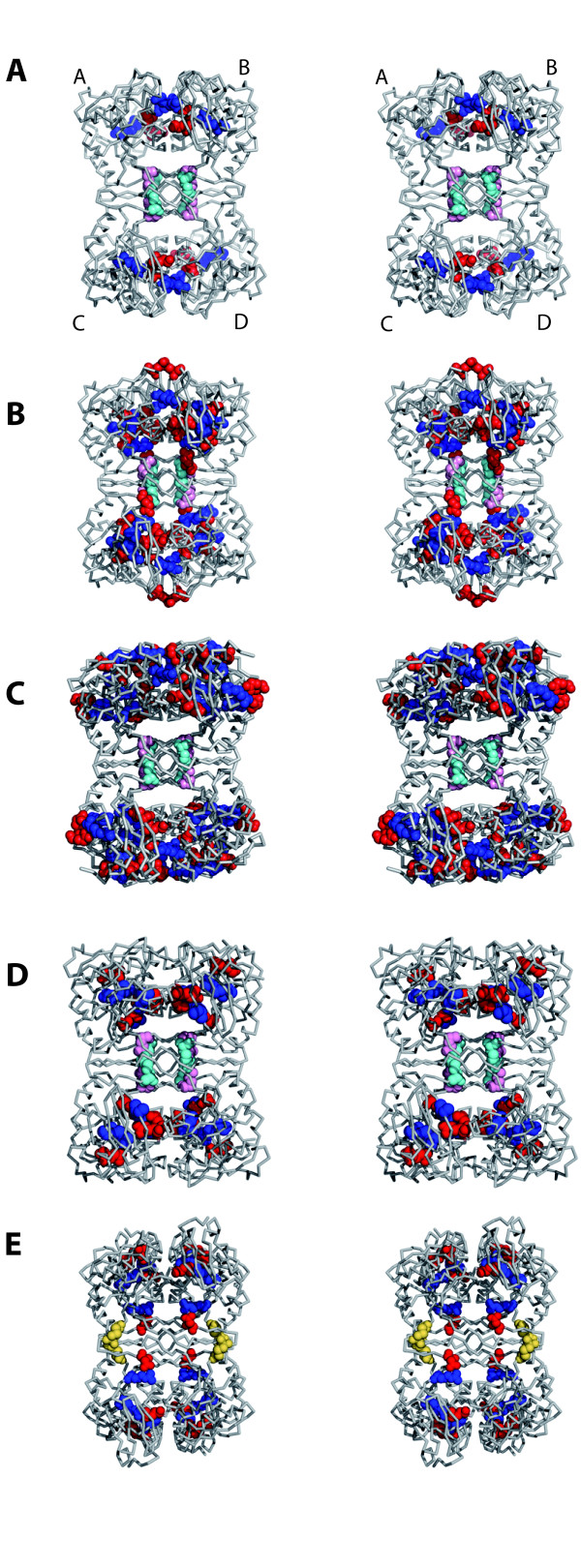
**Stereo views highlighting the regions interacting across the dimeric and tetrameric interface**. The dimeric interfaces are between dimers AB and CD, and the tetrameric interfacebetween dimers AC et BD. Residues involved in ionic interactions at the A/B dimer interface are shown in red or blue spheres (for acidic or basic residues, respectively). Glu226 and Arg230, which form a salt bridge involved in the tetrameric assembly are shown in pink and cyan spheres, respectively. Inter-monomer disulfide bridges between cysteines 196 and 233 in _Pa_TrmI are shown in yellow. **A **_Mt_TrmI, **B **_Tt_TrmI, **C **_Tm_TrmI, **D **_Aa_TrmI, **E **_Pa_TrmI.

The architecture of the TrmI tetramer (Figure 1(B) and 4(C4) and Table 3) shows that the formation of the A/B dimer involves a large buried surface area, which correlates with a high number of residues present at the interface between monomers. The A/C dimer presents a buried surface area that is reduced by a factor around 4 compared to the A/B one and that involves much less residues at the interface. Therefore, TrmI proteins can be described as a dimer of tightly-assembled dimers (A/B and C/D). The A/C dimer, in all available TrmI structures, presents very similar numbers of contacts with about ten hydrogen bonds between the A and C monomers (Table 3). Most residues involved at this interface belong to the C-terminal β-strands (β6, βG and β7, Figures 1(C) and 2). Two charged residues (Glu226 and Arg230, _Tm_TrmI numbering) are involved in two conserved bidentate ionic interactions to form contacts specific of the tetramer at the A/B and C/D dimer interfaces in all TrmIs except _Pa_TrmI (Figures 2 &4). It was shown that suppressing this conserved ionic interaction by replacing both equivalent interacting residues in the TrmI-6 and TrmI-61 subunits of the *S. cerevisiae *enzyme by alanine did not prevent formation of the tetramer [23]. These mutations by themselves were not sufficient to destabilize the tetramer because other important interactions remained intact. In _Pa_TrmI, these ionic interactions are replaced essentially by four inter-monomer disulfide bridges (Figure 4(E)). Concerning the A/B interface of TrmIs, the comparison of the number of H-bonds does not indicate a trend that correlates with higher thermostability. Other interactions inside the tight A/B dimer are rather extensive and mainly ionic, particularly for _Tm_TrmI (Figure 4). For the A/B dimer, the number of ionic interactions at the interface between monomers A and B is clearly increased for TrmI proteins from thermophilic organisms (Table 3).

The dimeric (A/B and C/D) and tetrameric (A/D and B/C) interactions also involve, in all TrmIs, van der Waals and hydrophobic interactions (Table 4; Additional File 1, Figure S1B). Although thermophilic TrmIs do not contain more alanine and aromatic residues than TrmIs from mesophiles (Additional File 1, Table S3), a higher number of residues participates in van der Waals interactions. Interestingly, _Pa_TrmI exhibits unusually low hydrophobic *P*v values [44] for both the dimeric and tetrameric interface, which indicates specific hydrophobic spots. In particular, Phe225 makes hydrophobic contacts with Leu223 and Val242. This interaction substitutes, together with the inter-monomer disulfide bridges (see below), to the conserved ionic salt bridges involved in tetramer stabilization in all other TrmI proteins. Indeed, Phe225 occupies the position equivalent to Arg230 that conservatively establishes a bidentate ionic interaction with Glu226 (Figure 2 and Additional File 1, Figure S1B). _Aa_TrmI also exhibits a low hydrophobic P-value for the A/B interface. Therefore, increased hydrophobic interactions at the dimeric interface in the hyperthermophilic TrmIs could account for the fact that the number of ionic interactions is not increased compared to the mesophilic and thermophilic proteins.

In summary, whereas thermophilic TrmIs use a higher number of ionic interactions to stabilize the A/B interface, hyperthermophilic TrmIs display increased hydrophobic interactions. This is in agreement with other studies that conclude that ionic interactions stabilizing crucial areas of structure, together with increased hydrophobic packing, are the most common means for stabilizing proteins at high temperatures, particularly oligomeric proteins. For example, comparison of tetrameric malate dehydrogenases from thermophilic and mesophilic bacteria indicated that higher thermostability comes first from the presence of polar residues that form additional hydrogen bonds within each subunit and then with the use of charged residues to form additional ionic interactions along the dimer-dimer interface, as well as additional aromatic contacts at the monomer-monomer interface in each dimer [38]. Moreover, comparative structural analysis of various citrate synthases also showed that higher growth temperatures correlate with reinforced electrostatic interactions in the subunit interface, as well as a reduced exposure of hydrophobic surface [39]. Interestingly, _Tm_TrmI, which has an optimum growth temperature of 80°C (the limit temperature to distinguish a thermophilic and a hyperthermophilic organism), has the highest number both of salt bridges and van der Waals contacts at the A/B dimer interface (Table 3). Therefore, _Tm_TrmI displays the highest buried surface areas for the A/B and A/C dimers and seems to employ both strategies used by thermophilic and hyperthermophilic TrmI proteins to achieve thermostability.

#### Archaeal _Pa_TrmI displays very different tetrameric contacts compared to the bacterial enzymes: Intersubunit disulfide bridges stabilize the tetramer

In addition to enhanced hydrophobic interactions at the interfaces, the archaeal _Pa_TrmI further increases its thermostability through the use of intersubunit disulfide bridges [21,27]. In _Pa_TrmI, the subunits are more tightly bound than in TrmIs from thermophilic bacteria, as shown by the value of 41 kcal/mole for the free energy difference between the dissociated and associated states (Table 3), despite the fact that the buried surface areas of the dimers and tetramer are less extensive than anticipated for a hyperthermophilic organism. This increased stability of the _Pa_TrmI tetramer compared to that of the other TrmI proteins results from the presence of four intermolecular disulfide bonds between Cys196 and Cys233 from different subunits (Figure 4E). Disulfide bonds are extremely rare in intracellular proteins from mesophilic organisms, due to the reductive nature of the cytoplasm [45]. In contrast, their presence in several intracellular thermophilic proteins has been shown to increase the stability of the proteins from these organisms at extreme temperatures [46-49]. The presence of inter-subunit disulfide bonds in _Pa_TrmI is consistent with the presence, in this organism, of a specific disulfide oxidoreductase protein, which is usually involved in the formation of intramolecular disulfide bonds within intracellular proteins from thermophilic organisms [50]. To determine whether the inter-subunit disulfide bridges are important for the stability and function of _Pa_TrmI, the single and double mutant proteins, in which Cys196 or/and Cys233 were replaced by serine, were produced and purified [21]. Whereas both single mutants migrated as a mixture of monomers and dimers on SDS-PAGE under non-reducing conditions, the double mutant migrated as a monomer. Gel filtration chromatography indicated that the single mutants formed high molecular weight aggregates and that the double mutant behaved predominantly as a dimer. Differential scanning calorimetry experiments indicated that the melting temperature of the double mutant is lowered by 16.5°C compared to that of the wild-type enzyme [27]. Finally, the double mutant was completely inactivated after preincubation at 85°C for 30 min. Altogether, these experiments indicated that the intersubunit disulfide bridges are essential for the thermostability of the tetramer of _Pa_TrmI.

## Conclusions

In the present study, we aimed at performing a detailed structural analysis to investigate the structural mechanisms underlying stability in TrmIs from organisms spanning a large variety of optimal growth conditions. Our analysis of the different TrmI monomers, in terms of amino-acid composition, three-dimensional structure, hydrogen-bonding and ionic interactions, did not uncover clear hallmarks to explain the stability of the extremozymes. On the contrary, we identified structural differences between TrmIs from mesophiles, moderate or extreme thermophiles, in the compactness of their dimeric and tetrameric units and in the nature of the interactions between their monomers. Thermophilic TrmIs display tight packing at these interfaces, resulting in a slight increase of compactness upon multimerization. To investigate further this feature, we analyzed the contacts between monomers. First, the number of ionic interactions between monomers increases in the thermophilic TrmIs and seems to be one of the main factors providing thermostability. Secondly, the two hyperthermophilic TrmI proteins display dimeric interfaces with increased hydrophobic interactions. In addition, _Pa_TrmI from *P. abyssi*, which grows not only under extreme conditions of temperature but also under high pressure, possesses inter-subunit disulfide bridges that were shown to be essential for its thermostability [21,27]. Therefore, our analysis revealed that different molecular strategies have emerged to ensure strong interactions at the interfaces between monomers in order to preserve the tetrameric architecture of TrmI under extreme life conditions. The key challenge for TrmI extremozymes is thus to preserve the tetrameric architecture crucial for their catalytic activity.

## Methods

### Multiple sequence alignment

The multiple structure-based sequence alignment was done with SSM (Secondary-structure Matching, http://www.ebi.ac.uk/msd-srv/ssm/) [51] and visualized with the program JOY (http://tardis.nibio.go.jp/cgi-bin/joy/joy.cgi) [52]. SSM was also used to determine the root mean square deviations between the superposed structures.

### Volume and surface calculations

The program VOIDOO was used to calculate molecular protein volumes and cavities [53]. The molecular volumes of the proteins per se were calculated using a grid spacing of 1 Å and a 0 Å radius probe and the cavity volumes with a 1.4 Å radius probe. The SAM/SAH cofactors and water molecules were omitted from the calculations. Accessible surface areas were calculated using the program ASA (P. Alzari, personal communication).

### Structural analysis

The salt bridges and H-bonds within one monomer (less than 3.5 Å) were analyzed with HBOND (http://cib.cf.ocha.ac.jp/bitool/HBOND/). The H-bonds, ionic interactions and van der Waals contacts between monomers were analyzed by examining the structures graphically. The interface areas and stabilities of the tetramers were calculated with the program *PISA, *omitting the ligands (http://www.ebi.ac.uk/msd-srv/prot_int/pistart.html) [44]. Our analysis was done using the PDB coordinates. However, several side chain atoms are not observed in the electron density and are therefore missing in some PDB files. Those missing residues could influence the contraction upon dimerization and tetramerization if they were located at the interface between the different subunits. This is not the case except for one side chain (Leu228 in *T. maritima *1O54). All other missing side chains belong to Lys, Arg, Glu and Gln residues at the surface of the protein, pointing towards the solvent.

The abbreviations used are: MTase: methyltransferase; rmsd: root mean square deviation; SAM: S-adenosyl-L-methionine; SAH: S-adenosyl-L-homocysteine; _Pa_TrmI: *Pyroccocus abyssi *tRNA m^1^A58 methyltransferase; _Tt_TrmI: *Thermus thermophilus *tRNA m^1^A58 methyltransferase; _Mt_TrmI: *Mycobacterium tuberculosis *tRNA m^1^A58 methyltransferase; _Aa_TrmI: *Aquifex aeolicus *tRNA m^1^A58 methyltransferase; _Tm_TrmI: *Thermotoga maritima *tRNA m^1^A58 methyltransferase; _Hs_TrmI-61: Trm61 subunit of *Homo sapiens *tRNA m^1^A58 methyltransferase.

## Authors' contributions

BGP coordinated the project. AG and PB analyzed the structures. AG drew the figures. All authors analyzed and interpreted the data. All authors wrote the manuscript. All authors discussed the results and approved the final manuscript.

## Supplementary Material

Additional file 1 **Table S1: Sequence identity of the different TrmI proteins**. Click here for file
