# Supplementary material for: Structural comparison of tRNA m1A58 methyltransferases revealed different molecular strategies to maintain their oligomeric architecture under extreme conditions
Source: BMC Struct Biol. 2011 Dec 14;11:48. doi: 10.1186/1472-6807-11-48 (PMC3281791; doi:10.1186/1472-6807-11-48)

Additional data

Table S1: Sequence identity of the different TrmI proteins.

	M. tuberculosis	*T. thermophilus*	*T. maritima*	*A. aeolicus*	*P. abyssi*
H. sapiens	0.35	0.37	0.30	0.30	0.30
M. tuberculosis		0.41	0.29	0.28	0.36
T. thermophilus			0.30	0.30	0.37
T. maritima				0.36	0.39
A. aeolicus					0.25

Table S2: Superposition of TrmI proteins. The first figure is the overall rmsd (Å) and the second figure the SSM Q-score[1]

A Superposition of the tetramers.

	T. thermophilus	*T. maritima*	*A. aeolicus*	*P. abyssi* (3MB5)
M. tuberculosis	1.9/ 0.52	1.7 / 0.53	2.8 / 0.39	2.2 / 0.46
T. thermophilus		1.8 / 0.53	2.4 / 0.48	2.8 / 0.41
T. maritima			2.3 / 0.46	2.2 / 0.46
A. aeolicus				3.2 / 0.36

B Superposition of the monomers.

	M. tuberculosis	*T. thermophilus*	*T. maritima*	*A. aeolicus*	*P. abyssi*
H. sapiens	1.6 / 0.54	1.6 / 0.58	1.7 / 0.53	1.7 / 0.57	2.3 / 0.45
M. tuberculosis		1.3 / 0.59	1.4 / 0.55	2.0 / 0.50	1.8 / 0.52
T. thermophilus			1.5 / 0.57	1.8 / 0.55	2.3 / 0.47
T. maritima				1.7 / 0.54	2.1 / 0.48
A. aeolicus					2.7 / 0.41

[1]The SSM Q score takes into account the number of aligned residues, their r.m.s.d. and the size of the proteins; a high Q score means a good similarity.

Table S3: Sequence comparison of the different TrmI proteins.

organism	number of alanine	number of proline	number of aromatics (HFWY)
H. sapiens	17 (5.1%)	15 (4.5%)	42 (12.5%)
M. tuberculosis	33 (11.8%)	17 (6.1%)	24 (8.6%)
T. thermophilus	29 (11.4%)	16 (6.3%)	34 (13.3%)
T. maritima	16 (6.1%)	12 (4.6%)	25 (9.5%)
A. aeolicus	13 (5.2%)	9 (3.6%)	37 (14.9%)
P. abyssi	17 (6.7%)	12 (4.7%)	31 (12.3%)

Table S4: **Number of H-bonds and ionic interactions in TrmI monomers.**

	Number of salt bridges per monomer (bidentate)	Number of H-bonds per monomer	Total electrostatic interactions per monomer
H. sapiens	11 (1)	203	214
M. tuberculosis	14 (3)	222	236
T. thermophilus	6 (0)	213	219
T. maritima	13 (2)	231	244
A. aeolicus	18 (1)	205	223
P. abyssi	20 (2)	211	231

Figure S1: Stereoviews of the interactions at the A/C dimer interface in $_{Aa}$TrmI. Chain A is labeled in black and chain C in pink. **A** H-bonds. **B** Salt-bridges and hydrophobic interactions.

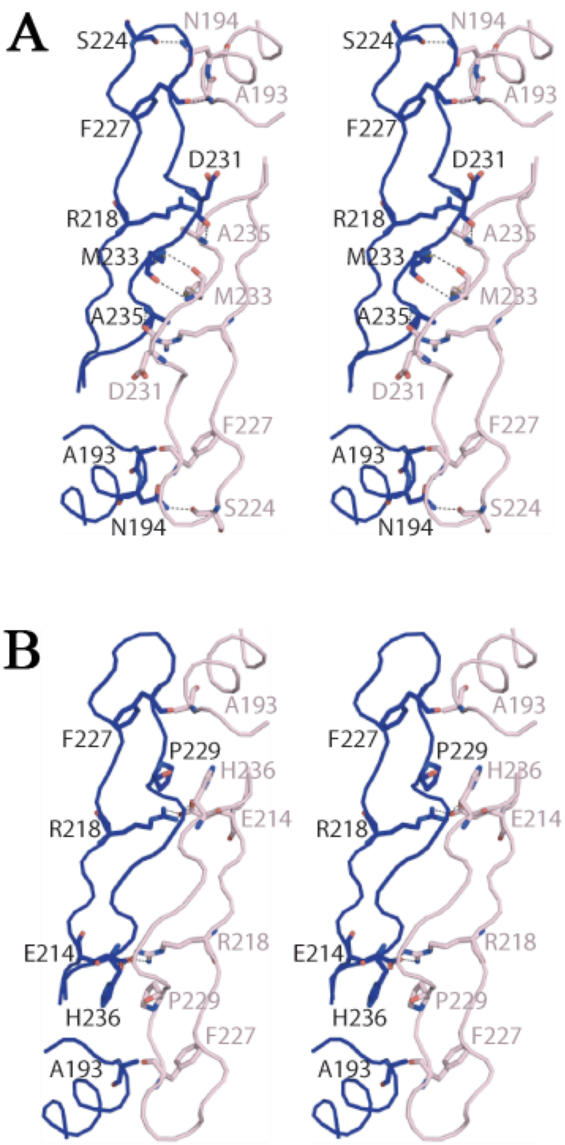

Supplement: Additional file 1 — Table S1: Sequence identity of the different TrmI proteins. [file 1472-6807-11-48-S1.PDF]
